# Optical Limiting Using the Two-Photon Absorption Electrical Modulation Effect in HgCdTe Photodiode

**DOI:** 10.1155/2013/245310

**Published:** 2013-10-01

**Authors:** Haoyang Cui, Junjie Yang, Jundong Zeng, Zhong Tang

**Affiliations:** School of Electronic and Information Engineering, Shanghai University of Electric Power, 2103 Pingliang Road, Shanghai 200090, China

## Abstract

The electrical modulation properties of the output intensity of two-photon absorption (TPA) pumping were analyzed in this paper. The frequency dispersion dependence of TPA and the electric field dependence of TPA were calculated using Wherrett theory model and Garcia theory model, respectively. Both predicted a dramatic variation of TPA coefficient which was attributed into the increasing of the transition rate. The output intensity of the laser pulse propagation in the pn junction device was calculated by using function-transfer method. It shows that the output intensity increases nonlinearly with increasing intensity of incident light and eventually reaches saturation. The output saturation intensity depends on the electric field strength; the greater the electric field, the smaller the output intensity. Consequently, the clamped saturation intensity can be controlled by the electric field. The prior advantage of electrical modulation is that the TPA can be varied extremely continuously, thus adjusting the output intensity in a wide range. This large change provides a manipulate method to control steady output intensity of TPA by adjusting electric field.

## 1. Introduction

Two-photon absorption (TPA) is a third-order nonlinear absorption process which is closely related to the imaginary part of nonlinear susceptibility of the material [1]. Because TPA has different selection rules than one-photon absorption (OPA), it is widely used in the spectroscopy analyzing of semiconductor [2]. Moreover, two-photon absorption (TPA) is an effective process to fulfill the nonlinear optical devices [3] owing to its high transparency at low incident intensity while blocking the transmission at high intensities. Although some theoretical investigations [4, 5] and experimental measurements [6] about TPA properties have been reported early, the constant electric field effects of TPA on the transmission intensity have not received much attention. This is surprising considering that strong fields are often present in semiconductor-based photonic devices, such as pn junction photovoltaic device. The lack of research is detrimental to the application of nonlinear optical devices.

In this paper, the dispersion dependence of TPA on photon frequency is calculated using second-order perturbation theory. Then, the electric field modulation effect of two-photon absorption coefficient (TPAC) in HgCdTe photodiode is simulated from a two-band model. The results show that the TPAC in space charge region (SCR) is enhanced dramatically by the build-in electric field. A TPA Franz-Keldysh Effect (FKE) mentioned by Garcia is adapted to interpret this phenomenon. On this basis, the dependence of output intensity on electric field is calculated using the TPA modulation relationship. It shows that the output intensity can be manipulated continuously by adjusting the bias voltage.

## 2. Theory Model

### 2.1. Frequency Dispersion Dependence of TPAC

The two-photon process where the two-photon transition occurs from an initial state |*i*〉 to a final state |*f*〉 involving simultaneous absorption of two photons was theoretically predicted by Göppert-Mayer [7] in 1931. Since the transition probability is proportional to *δ*(*E*_*f*_ − *E*_*i*_ − *ℏω*_1_ − *ℏω*_2_), it may be obtained from Fermi golden rule:

(1) $$\begin{array}{c} W^{\left(2\right)}=\frac{2\pi }{\hbar }\sum {\left|M_{cv}^{\left(2\right)}\right|}^{2}\delta \left(E_{c}\left({\vec{k}}_{c}\right)-E_{v}\left({\vec{k}}_{v}\right)-2\hbar \omega \right), \end{array}$$

where $\vec{k}$ is the wave vector of the internal motion of the exciting and *M*_*cv*_^(2)^ is the transition matrix element, respectively. In order to solve (1), the energy band, momentum matrix element, and the intermediate state should be approximated to simplify the calculation. However, the approximations and simplifying assumptions used in this process would result in a large discrepancy between the calculation and experiment results of TPAC. In the computing models presented in the literatures, Wherrett [8] model has been generally recognized. From the second-order perturbation theory, the interaction matrix element *H*_*fi*_′ between initial state |*i*〉 and final state |*f*〉 associated with the absorption of one photon can be obtained as:

(2) $$\begin{array}{c} M_{cv}^{\left(2\right)}=\frac{\sum_{i}H_{ci}^{\prime }H_{iv}^{\prime }}{E_{iv}\left(\vec{k}\right)-\hbar \omega },\\ H_{fi}^{\prime }=\frac{q}{im\omega }{\left(\frac{2\pi I}{nc}\right)}^{1/2}\varepsilon \cdot p_{fi}, \end{array}$$

where *n*, *p*_*fi*_, and *ε* are refractive index, momentum matrix element, and radiation polarization, respectively. For the spin degeneracy for the bands, the TPA transition rate *W* ^(2)^ can be written as

(3) W(2)=1πℏ[(2mcvℏ2)3/2(2ℏω−Ec−Ev)1/2] ×[(qmw)4(2πInc)2    ×〈|Scv(2)((2mcv)1/2(2ℏω−Ec−Ev)1/2ℏ)|2〉],

where *S*_*cv*_^(2)^ = ∑_*i*_ *p*_*ci*_ *p*_*iv*_[*E*_*iv*_((2*m*_*cv*_)^1/2^(2*ℏω*−*E*_*c*_−*E*_*v*_)^1/2^/*ℏ*) − *ℏω* ]^−1^ and *p* represents *ε* · *p*. Retaining the matrix element corresponding to the direct contribution, TPAC can be expressed as

(4) β(ω)=292π(q2ℏc)2f2  fℏPn2Eg3(2ℏω/Eg−1)3/2(2ℏω/Eg)5=A(2ℏω/Eg−1)3/2(2ℏω/Eg)5,

where *c*, *q*, *E*_*g*_ and *ℏω* are speed of sound, electron charge, energy gap, and incident photon energy, respectively. *P* ∝ *p*_*cv*_ *ℏ*/*m* is Kane momentum parameters. *f* is defined as the numerical factor. Equation (4) shows the frequency dispersion relation of TPAC.

**Figure 1:**
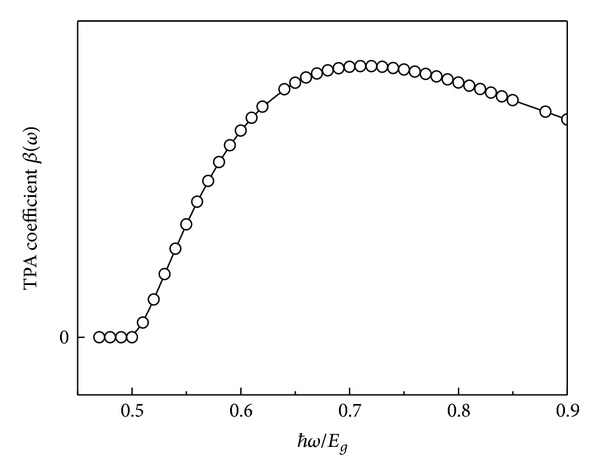
The calculating frequency dependence of TPAC and incident photon energy.

Figure 1 shows the frequency dispersion relation of TPA. It can be seen that the TPAC is almost equal to zero for an incident photon energy less than half energy gap; this shows that no transition occurs between energy bands. The TPAC will continuously increase with the photon energy when the photon energy is larger than half energy gap. Then, the TPAC will show a decreasing tendency if the photon energy increased more. This indicates that there is a maximum value in the TPAC. The effects of the external electric field on TPAC have been more complicated; in the electric field, the problem is further complicated and will be discussed below.

### 2.2. Electric Field Dependence of TPAC

A two-band model consisting of heavy-hole and light-hole bands mentioned by Garcia [4] is used to discuss the dependence of TPAC on electric field here. Using a nonparabolic approximation of the state's density, the impact of electric field intensity on the TPA transition probabilities can be derived from the Fermi golden rule [4]:

(5) W2=1(2π)2∑n∑n′∫dkW(2)(k),W(2)(k)=|Pvc|22πℏ(2πmcωNmk·z^)2(ℏ22MFe)2/3×|IN(ek·z^A0mcωc)|2[Ai(δ2)ancan′v]2× δ(Ek⊥c+Ek′⊥v+Ee−Eh+Eg−2ℏω),

where *P*_*vc*_, *F*, *Ai*(*z*), *m*_*c*_, *m*_*v*_, and *m*_*u*_ are interband momentum matrix element, electric field, Airy function, effective mass of electron and hole, and the reduced mass, respectively. In the whole excitation process, principle of energy conservation is satisfied by *δ* function, while momentum conservation is satisfied by Δ*k* = *k*_*c*_ − *k*_*v*_ = 0; thus the TPAC can be derived as

(6) β(2)(ω,F)=(5π23)K(EpEgEμ)1/2n2(2ℏω)5 ×{2[ε02Ai2(ς0)−ς0|Ai′(ς0)|2]   −Ai(ς0)Ai′(ς0)},

where *ς*_0_ = (2*ℏω* − *E*_*g*_)/*E*_*μ*_, *E*_*μ*_ = (*ℏ* ^2^ *e* ^2^ *F* ^2^/2*m*_*μ*_)^1/3^, is the characteristic energy of the dc electric field. Because of the introduction of this parameter, the TPAC will change with the electric field strength. *E*_*p*_ is the transition matrix. *K* = 1940 cm/GW (eV) is a material-independent constant.

**Figure 2:**
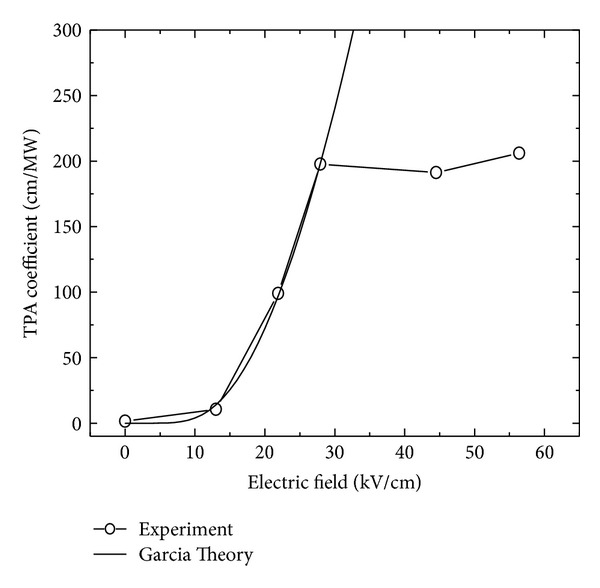
The dependence of TPAC on the build-in electric field strength. The circle points are the experimental data, and the line represents the calculation data.

Using the expression in (6), the TPAC at different electric field is simulated (shown in Figure 2). We compared the simulation results with the two-photon absorption experimental results of HgCdTe photodiode [6]. The HgCdTe photodiode used in this work was prepared by the literature methods [6, 9–12]. As one can see, the calculated and the experimental data have a similar dependence on the electric field at 0 < *F* < 30 kV/cm. However, if the electric field *F* > 30 kV/cm, the calculation data still increase, while the experimental TPAC shows saturation. This can be attributed to the saturation of the electric field in the pn junction in this range, and the electric field strength in the SCR will not increase anymore.

Figure 2 shows that the TPAC at 0 V (*β*_1_ = 10.5 cm/MW) has been enhanced by about seven times comparing to zero electric field (*β*_0_ = 1.5 cm/MW). When the built-in electric field varies from 13.4 kV/cm to 30 kV/cm, TPAC will be enhanced into 198 cm/MW, about 19 times increasing. This significant enhancement is attributed to the FEK of the TPAC in SCR. The absorption coefficient increased in the strong electric field of SCR by increasing the coupling intensity of electron and hole wave functions. FKE occurs because the electric field assisted tunneling process in SCR makes the effective energy gap shrink for an electron in the interband transition. The band gap shrinkage is proportional to the electric field [13] as Δ*E*_*g*_ ~ *F*. Consequently, the electrons exited from valance band have more probability to transmit into the conduction band. This mechanism has made the TPAC within the SCR increase [6, 14].

### 2.3. Electric Field Dependence of Output Intensity

**Figure 3:**
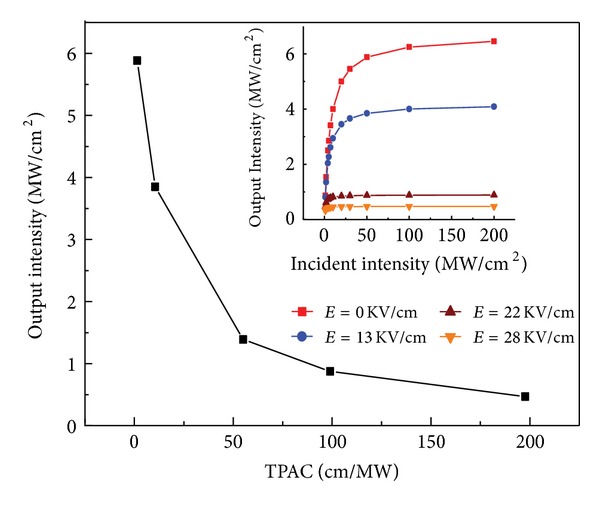
The dependence of output intensity on TPAC when the incident intensity is 50 MW/cm^2^. The insert is the dependence of output intensity on incident intensity at different electric field.

Based on the analysis given above, it can be known that the TPAC can be adjusted effectively by varying the electric field applied, thus changing the output light intensity. Undoubtedly, this TPAC manipulating effect is very attractive for the nonlinear optical devices such as optical limiter. In the following, we will simulate the effects of TPAC manipulation on the output intensity. The output intensity of pn junction can be solved using function-transfer method [15]:

(7) $$\begin{array}{c} I_{n+1}\approx I_{n}-\frac{\beta I_{n}^{2}\Delta x}{\left(1+\beta I_{n}\Delta x\right)}, \end{array}$$

where Δ*x* is the cell thickness of absorption layer and *I*_*n*_ is the incident intensity of *n* layer. As a result of back-illuminated, the incident light passes through the flat band of *p* region first and then into the SCR. Comparing with the thick base region, the thickness of the heavy doped emitter can be neglected. Therefore, only the optical loss in flat belt layer and SCR is considered here. Using the conclusion of TPAC modulation mentioned above, the effects of TPAC manipulation on the output intensity can be simulated, which is shown in Figure 3.

It can be seen from Figure 3 that, with the TPAC increasing, the output intensity will decrease dramatically. This can be attributed the enhancement of TPA in SCR, resulting in more carriers that are excited by the photons, and the output intensity transmitted from material is weakened continually. The inset of Figure 3 shows that the output intensity increases non-linearly with increasing intensity of incident light and eventually reaches saturation. The output saturation intensity depends on the electric field strength the greater the electric field, the smaller the output intensity. This shows that the clamped saturation intensity can be controlled by the electric field. 

## 3. Conclusion

In conclusion, we calculated the dependence of electric field on the TPAC and the output intensity in pn junction photodiode. Because of the TPA FKE, the TPAC could be manipulated by adjusting the electric field within the SCR. We simulated the dependence of electric field strength on the output intensity. It shows that the clamped saturation intensity can be controlled by the electric field. From the analysis of nonlinear TPA in the semiconductor, one can obtain a method to control steady output intensity of TPA pumping by adjusting electric field or bias voltage. Since the TPA is an important mechanism in semiconductors, our initial result on the electric field induced tunability of TPA, and its influence on the output intensity would can be very useful for the nonlinear optical device.
